# Supplementary material for: Oral health practices and oral hygiene status as indicators of suicidal ideation among adolescents in Southwest Nigeria
Source: PLoS One. 2021 Feb 25;16(2):e0247073. doi: 10.1371/journal.pone.0247073 (PMC7906320; doi:10.1371/journal.pone.0247073)
Supplement: S2 File — (DOCX) [file pone.0247073.s002.docx]

**Sexual Abuse, Mental Health and Oral Health Survey, NIGERIA**

# INDIVIDUAL INTERVIEW SCHEDULE FOR ADOLESCENTS AGED 10-19 YEARS

QUESTIONNAIRE IDENTIFICATION NUMBER |___|___|___|___|___|___|

**Introduction:** My name is…… ……… We are interviewing adolescents here in [**NAME OF CITY, TOWN OR SITE**] in order to find out about certain behaviors that affect their health and risk for HIV infection in this environment.

**Confidentiality and consent:** I am going to ask you questions some of which may be very personal. Your answers are completely confidential. Your name will not be written on this form, and will never be used in connection with any of the information you tell me. You may want to know that this exercise is taking place all other states in Nigeria (Lagos, Kano, Oyo, Enugu, Plateau, xxx, xxx). It involves xxx adolescents from these states. Your honest answers to these questions will help us better understand what adolescent needs with respect to their oral, mental and sexual health. The information collected from you and people like you will help us make recommendations to the government on how to address these needs we identify through this research. We would greatly appreciate your help in responding to this survey.

(Signature of interviewer certifying that informed consent has been given verbally by respondent)

Interviewer visit

|  | **Visit 1** | **Visit 2** | **Visit 3** |
| --- | --- | --- | --- |
| Date |  |  |  |
| Result |  |  |  |
| Interviewer |  |  |  |
| Street Address |  | | |

**001 INTERVIEWERS: Code [____|____]**___| **Name__________________________**

**Signature__________**

**002 DATE OF INTERVIEW:___\ ____ \ _____ TIME INTERVIEW STARTED_______________**

**DD MM YYYY**

**CHECKED BY SUPERVISOR__________________CODE[__]__]__] Date ____________________**

Name of Coder____________________|___|___| Signature________________ Date______________.

Section 1: Background characteristics

| **No.** | **Questions and filters** | **Coding categories** | **Skip to** |
| --- | --- | --- | --- |
| Q101 | **[RECORD SEX OF THE RESPONDENT]** | Male…………….1  Female………….2 |  |
| Q102 | In what month and year were you born? | Month [___\|___]  Don’t know month ………..88  Year [___\|___ [___\|___]  Don’t know year ………..88 |  |
| Q103 | How old were you as at your last birthday?  **[COMPARE WITH Q102 IF NEEDED AND CORRECT Q103]** | Age in completed years [___\|___] |  |
| Q104 | What is your occupation i.e. what kind of work do you mainly do? | Director/upper management…………….……1  Other management……………………….……2  Sales manager/representative/Insurance Broker..3  Professional/Specialist…………………………4  Self employed/Own small business…………….5  Self employed (informal sector /hawkers/vendors etc.)…………...6  Blue collar skilled & semi skilled……………….7  Unskilled……………………………………....8  Clerk/clerical…………………………………..9  Civil Servant…………………………………10  Farmer/Forestry/Fishing/Mining……………11  Housewife……………………………………12  Pensioner/Retired……………………………13  Unemployed…………………………………14  Student……………………………………….15  Others specify[ ]…16 |  |
| Q105 | Have you ever attended school? | Yes………………….. ….. 1  No…………………. ….. 2 | **→Go to**  **Q107** |
| Q106 | What is the highest level of school you attended: Quranic only, primary, secondary or higher? | Quranic only………1  Primary …….. …..2  Secondary …….….. 3  Higher ………….. 4 |  |
| Q106A | What is the class/form/year you are currently or completed when you were in school? | Class (Primary) [___]  Form (Secondary) [___]  Year (Tertiary) [___]  Others [___] |  |
| Q107 | What is your religion? | Islam…………. 1  Protestant.……. 2  Catholic……….. 3  Traditional.…….. 4  No religion ……. 5  Others specify.[ ]…6  No Response………9 |  |
| Q108 | How old is your mother at her last birthday?  (please ask directly from parents) | Age in completed years [___\|___] |  |
| Q109a | What is the occupation of your mother i.e. what kind of work do you mainly do?  (please ask directly from parents) | Director/upper management…………….……1  Other management……………………….……2  Sales manager/representative/Insurance Broker..3  Professional/Specialist…………………………4  Self employed/Own small business…………….5  Self employed (informal sector /hawkers/vendors etc.)…………...6  Blue collar skilled & semi skilled……………….7  Unskilled……………………………………....8  Clerk/clerical…………………………………..9  Civil Servant…………………………………10  Farmer/Forestry/Fishing/Mining……………11  Housewife……………………………………12  Pensioner/Retired……………………………13  Unemployed…………………………………14  Student……………………………………….15  Others specify[ ]…16 |  |
| Q109B | What is the highest level of school your mother attended: Quranic only, primary, secondary or higher?  (please ask directly from parents) | Quranic only………1  Primary …….. …..2  Secondary …….….. 3  Higher ………….. 4 |  |
| Q109C | What is the class/form/year your mother completed when she was in in school?  (please ask directly from parents) | Class (Primary) [___]  Form (Secondary) [___]  Year (Tertiary) [___]  Others [___] |  |
| Q110 | How old is your Father at his last birthday?  (please ask directly from parents) | Age in completed years [___\|___] |  |
| Q110a | What is the occupation of your father i.e. what kind of work do you mainly do?  (please ask directly from parents) | Director/upper management…………….……1  Other management……………………….……2  Sales manager/representative/Insurance Broker..3  Professional/Specialist…………………………4  Self employed/Own small business…………….5  Self employed (informal sector /hawkers/vendors etc.)…………...6  Blue collar skilled & semi skilled……………….7  Unskilled……………………………………....8  Clerk/clerical…………………………………..9  Civil Servant…………………………………10  Farmer/Forestry/Fishing/Mining……………11  Housewife……………………………………12  Pensioner/Retired……………………………13  Unemployed…………………………………14  Student……………………………………….15  Others specify[ ]…16 |  |
| Q110B | What is the highest level of school your father attended: Quranic only, primary, secondary or higher?  (please ask directly from parents) | Quranic only………1  Primary …….. …..2  Secondary …….….. 3  Higher ………….. 4 |  |
| Q110C | What is the class/form/year your father completed when she was in in school?  (please ask directly from parents) | Class (Primary) [___]  Form (Secondary) [___]  Year (Tertiary) [___]  Others [___] |  |
| Q111 | To which ethnic group do you belong? | Birom ………………………………..1  Bura ………………………………….2  Edo…………………………………...3  Efik…………………………………. 4  Fulani …………………………...……5  Gwari…………………………….……6  Hausa…………………………….……7  Ibibio……………………………….…8  Idoma…………………………….….9  Igala……………………………..…….10  Igbo…………………………….…….11  Ijaw …………………………………..12  Ikwere…………………………...……13  Itsekiri………………………………..14  Kaje………………………………….15  Kanuri………………………….……16  Okrika……………………………….17  Nupe …………………………..……18  Shuwa-Arab…………………………19  Urhobo………………………………20  Tiv…………………………….……..21  Yoruba……………………………….22  Others specify[ ]..23 |  |
| Q112 | Who are you currently living with | Both Parents ……………………1  Mother only………………2  Father only………………3  Mother and stepfather …………4  Father and step mother……………5  Guardian………………….6  Cohabiting ………………….7  Room mates ………………….8  Other [ ………………] -9 |  |
| Q113 | How many of you are living in your home? | 1  2  3  4  5  6  Others…………………….99 |  |
| Q114 | How many children do your parents have? | 1  2  3  4  Others…………………….99 |  |
| Q115 | What is your position among the children your parents have? | 1  2  3  4  Others…………………….99 |  |
| Q116 | How many meals do you eat per day?  [READ OUT OPTIONS]  [SINGLE CODE ONLY] | Cannot guarantee one meal a day throughout the month…1  Only afford one meal a day throughout the month. 2  Only afford two meals a day throughout the month3  Afford three meals a day throughout the month…4  No Response …………………………………….5 |  |

Section 2: Sexual History: Number and Types of partners

| **No.** | **Questions and filters** | | **Coding categories** | **Skip to** |
| --- | --- | --- | --- | --- |
|  | **[TELL THE RESPONDENT]**  **I need to ask you some personal questions about sexual activity in order to gain a better understanding of some family life issues.** | | |  |
| Q201 | At what age did you first have sexual intercourse if ever? | Age in years [___\|___]  Never…………….87  Can’t remember…….. 88  No Response………99 | | **→Go to Q501** |
| Q201A | What was the circumstances around your first sexual intercourse | In love………… ….... 1  Having fun ……………… 2  Peer pressure……….3  To obtain money………… ….... 4  Forced ……………… 5  Others [___________________]…..6  No response……….9 | |  |
| Q201B | Have you ever had sex in exchange for money/ favours or gifts? | Yes………… ….... 1  No ……………… 2  No response……….9 | |  |
| Q202 | Surveys reveal that many people have had more than one sexual partner at the same time. Would you say this has ever happened to you? | Yes …………….. 1  No …………..… 2 | |  |
| Q203 | Have you had sexual intercourse in the last 12 months? | Yes …………….. 1  No …………….. 2  No Response……9 | | **→Go to**  **Q501** |
| Q203A | There different other forms of sexual activities. Which of these activities are you aware of? | Anal sex ………… ….... 1  Oral sex ……………… 2  Vagina sex ……………… 3  Others (specify)............. 4  None .................. 5  No response……….9 | |  |
| Q203B | Which of these forms of sex have you ever been involved in? | \| Anal \| Yes............ \| 1 \| \| --- \| --- \| --- \| \| No............ \| 2 \| \| Oral \| Yes............ \| 3 \| \| No............ \| 4 \| \| Vagina \| Yes............ \| 5 \| \| No............ \| 6 \| \| Others \| Yes............ \| 7 \| \|  \| No............ \| 8 \| | | **If No to all, Skip to Q404** |
| Q203C | Which one of these forms of sex have you been involved in (in the last 12 months)? | \| Anal \| Yes............ \| 1 \| \| --- \| --- \| --- \| \| No............ \| 2 \| \| Oral \| Yes............ \| 3 \| \| No............ \| 4 \| \| Vagina \| Yes............ \| 5 \| \| No............ \| 6 \| \| Others \| Yes............ \| 7 \| \|  \| No............ \| 8 \| | | **If No to all, Skip to Q404** |
| Q203D | Think about the persons you have had sex with through the anus in the last 12 months.    How many were:   1. Your spouse(s) or partners who you were living together with 2. Boy/girl friends 3. Partners with whom you had commercial sex 4. Partners you met on a casual basis   E Female  F Male  **[IF NONE FOR ANY OF THE PARTNER TYPES CODE ‘00’]** | **MARITAL OR LIVING TOGETHER**  [___\|___]  **BOY/GIRLFRIEND**  [___\|___]  **COMMERCIAL** [___\|___]  **CASUAL** [___\|___]  **WOMEN** [___\|___]  **MEN**  [___\|___]  No Response……99 | |  |
| Q203E | The last time you had sex through the anus did you use a condom? | Yes………… ….... 1  No ……………… 2  No response……….9 | |  |
| Q204 | How many sexual partners have you had in the past 12 months? | **NUMBER**  [__]__]  No Response……..99 | |  |
| Q205 | Think about the persons you have had vagina, oral or other forms of sex with in the last 12 months.  How many were:   - Your spouse(s)/partners who you were living together with - Boy/girl friends - Partners with whom you had commercial sex - Partners you met on a casual basis   **[IF NONE FOR ANY OF THE PARTNER TYPES CODE ‘00’]** | **MARITAL OR**  **LIVING TOGETHER**  [___\|___]  **BOY/GIRLFRIEND**  [___\|___]  **COMMERCIAL** [___\|___]  **CASUAL** [___\|___]  No Response……99 | |  |
| Q205A | **CHECK Q903A. DID RESPONDENT HAVE VAGINA SEX**  Think of your **very last vagina sex act** In that **very last sex act,** was a condom used? | Yes………1  No……….2  No response……….9 | |  |
| Q205B | **CHECK Q903A. DID RESPONDENT HAVE ANAL SEX**  Think of your **very last anal sex act** In that **very last sex act,** was a condom used? | Yes………1  No……….2  No response……….9 | |  |
| Q205C | **DID RESPONDENT HAVE ANAL SEX**  We are aware that people have various sexual preferences. No sexual preference is right or wrong. Think of your **very last anal sex act.** Who did you have anal sex with? | Male………1  Female……….2  No response……….9 | |  |
| Q205D | **CHECK Q903A. DID RESPONDENT HAVE ORAL SEX**  Think of your **very last oral sex act.** In that **very last sex act,** was a condom or dental dam used? | Yes………1  No……….2  No response……….9 | |  |
| Q205E | Once again, we are aware that people have various sexual preferences. No sexual preference is right or wrong. We will like to know who you had your last oral sex act with? | Male………1  Female……….2  No response……….9 | |  |
| Q206 | **CHECK Q905. DID RESPONDENT HAVE SEX WITH BOY/GIRLFRIEND AND/OR CASUAL PARTNER AND/OR COMERCIAL SEX PARTNER? Y/N→**  **↓** | → → → | | **→Go to Q910** |
| Q207 | Think of your **very last sex act** with a non-marital, non-cohabiting partner. In that **very last sex act,** was a condom used? | Yes………1  No……….2 | | **→Go to Q908** |
| Q207A | What was the **MAIN** reason why you used a condom that time? Was it …..  **[READ OUT]** | For protection from HIV/STIs……1  To prevent unwanted pregnancy….2  For protection from both HIV/STIs and unwanted pregnancy…..3  Others specify[ ]…4 | |  |
| Q208 | This partner with whom you had your last sex act, was he/she younger, about the same age or older than you? | Younger….1  About the same age……2  Older than me…….3 | | **→ Go to Q909A**  **→ Go to Q910** |
| Q209 | (If older,) do you think he/she was less than 10 years, or 10 or more years older than you? | Less than 10 years older……1  10 or more years older….2  Don’t know the difference….8 | | **Go to**  **Q910** |
| Q209A | (If younger,) do you think he/she was less than 10 years, or 10 or more years younger than you? | Less than 10 years younger……1  10 or more years younger….2  Don’t know the difference….8 | |  |
| Q210 | **[ASK ALL WHO HAVE HAD SEX IN THE LAST 12 MONTHS IN Q403]**  How many sexual partners do you currently have including casual and commercial partners? | **Number**  [__]__]  No Response……..99 | |  |
| Q211 | Of all your current sexual partners, how many are your….  Spouse/ partners who you are living together with?  Non marital and non-cohabiting partners?  **[IF NONE TO ANY CODE ‘00’]** | **Number**  [___\|___]  No Response…….99  **Number**  [___\|___]  No Response……..99 | |  |

Section 3: History of Sexual Assault

| **No.** | **Questions and filters** | **Coding categories** | **Skip to** |
| --- | --- | --- | --- |
| Q301 | How old were you at the age of your first assault? | Age [___\|___] |  |
| Q302 | What was the route of sexual assault (tick as many as applies) | Oral ………..1  Vagina………..2  Anal ………..3  Cannot remember­………..88  None of the above………..99 |  |
| Q303 | Did you tell anyone? | Yes ………..1  No ………..2  No response ………..99 | **→Go to**  **Q301** |
| Q303a | If yes, how old were you told someone about the assault? | Age [___\|___] |  |
| Q303b | What was the response? |  |  |
| Q303c | Who were you living with at the time of the first assault | Both Parents ……………………1  Mother only………………2  Father only………………3  Mother and stepfather …………4  Father and step mother……………5  Guardian………………….6  Cohabiting ………………….7  Room mates ………………….8  Other [………………] -9 |  |
| Q303c | Have you experienced another assault? | Yes ………..1  No ………..2  No response ………..99 |  |
| Q303d | How many times have you experienced other sexual assaults? | Number [___\|___] |  |
| Q303e | What was/were the route of sexual assault(s)  *(tick as many as applies)* | Oral ………..1  Vagina………..2  Anal ………..3  Cannot remember­………..88  None of the above………..99 |  |
| Q303f | How old were you when you experienced a second assault? | Age [___\|___] |  |

**Section 4: Knowledge of caries prevention**

| No. | **Questions and filters** | Coding categories | Skip to |
| --- | --- | --- | --- |
| I will be asking you a few questions about what you know on how to care for oral health. All your responses are confidential. I will be depending on your truthful statements to make plans for planning for children in this environment. Please do not guess. Also, do feel free to ask about things you do not understand. Thank you. | | | |

| Q401 | Fluoridation of drinking water is an effective, safe, and efficient way to prevent holes from forming on the teeth | Strongly agree………….….1  Agree………….….2  Disagree………….….3  Strongly disagree………….….4  Don’t know………….….5 | |  |
| --- | --- | --- | --- | --- |
| Q402 | Use of fluoride containing toothpaste is an effective, safe, and efficient way to prevent holes from forming on the teeth. | Strongly agree………….….1  Agree………….….2  Disagree………….….3  Strongly disagree………….….4  Don’t know………….….5 | |  |
| Q403 | The number of times you eat sugar containing food has a great role in producing holes in the teeth | Strongly agree………….….1  Agree………….….2  Disagree………….….3  Strongly disagree………….….4  Don’t know………….….5 | |  |
| Q404 | Fissure sealant is effective in the prevention of holes developing in newly erupted molars | Strongly agree………….….1  Agree………….….2  Disagree………….….3  Strongly disagree………….….4  Don’t know………….….5 | |  |
| Q405 | Rinsing teeth with a lower amount of water after tooth-brushing increases the effect of fluoride | Strongly agree………….….1  Agree………….….2  Disagree………….….3  Strongly disagree………….….4  Don’t know………….….5 | |  |
| Q406 | Using fluoride toothpaste is more important than the brushing *per se* for preventing holes from forming on the teeth. | Strongly agree………….….1  Agree………….….2  Disagree………….….3  Strongly disagree………….….4  Don’t know………….….5 | |  |
| Q407 | Brushing twice daily with fluoride containing toothpaste is effective for preventing holes from developing in the teeth | | Strongly agree………….….1  Agree………….….2  Disagree………….….3  Strongly disagree………….….4  Don’t know………….….5 |  |
| Q408 | It is important to visit the dental clinic regularly as a measure for preventing holes from forming in the teeth. | | Strongly agree………….….1  Agree………….….2  Disagree………….….3  Strongly disagree………….….4  Don’t know………….….5 |  |

**Section 5: Oral Health Behaviour**

| No. | **Questions and filters** | Coding categories | Skip to |
| --- | --- | --- | --- |

| Q501 | How often do you usually brush your teeth? | Irregularly or never………….….1  Once a week………….….2  A few (2-3) times a week………….….3  Once a day………….….4  Twice a day………….….5  More than twice a day………….….6  No response………….….99 | |  |
| --- | --- | --- | --- | --- |
| Q502 | How often do you use toothpaste containing fluoride when brushing? | Always………….….1  Quiet often………….….2  Seldom………….….3  Not at all………….….4  No response………….….99 | |  |
| Q503 | How often do you floss your teeth? | Irregularly or never………….….1  Once a week………….….2  A few (2-3) times a week………….….3  Once a day………….….4  Twice a day………….….5  More than twice a day………….….6  No response………….….99 | |  |
| Q504 | How often do you eat sugar-containing snacks or drinks between your main meals? | About 3 times a day or more ………….….1  About twice a day ………….….2  About once a day ………….….3  Occasionally; not every day ………….….4  Rarely or never eat between meals ………….….5  No response………….….99 | |  |
| Q505 | What do you do for your dental check-ups? | I go to a dentist to do that………….….1  I ask my or colleagues to do if for me………….….2  I do it myself………….….3  There is no need to attend dental check-ups………4  No response………….….99 | |  |
| Q506 | When was your last dental check-up? | Within the last 6 months ………….….1  More than 6 months to one year ago ………….….2  More than 1 to 2 years ago ………….….3  More than 2 to 5 years ago ………….….4  More than 5 years ago ………….….5  Never ………….….6  Do not remember ………….….88  No response………….….99 | |  |
| Q507 | Do you smoke cigarettes? | | No, never ………….….1  No, I used to, but I quit ………….….2  Yes, once a month or less ………….….3  Yes, a few times (2-3) a month ………….….4  Yes, a few times (2-3) a week ………….….5  Yes, once a day or more ………….….6  No response………….….99 |  |
| Q508 | Do you have any of these oral habits | | Digit an finger sucking ………….….1  Tongue sucking ………….….2  Tongue thrusting ………….….3  Lip sucking ………….….4  Lip biting ………….….5  Nail biting ………….….6  Object biting ………….….7  bruxism ………….….8  No response………….….99 |  |

**Section 6: History of dental service utilization**

| No. | **Questions and filters** | Coding categories | Skip to |
| --- | --- | --- | --- |

| Q601 | What has inhibited you from visiting dentists in the past? | No oral health problem………….….1  No availability of funds………….….2  Do not know where a dental clinic is………….….3  Feeling of lack of control with dentist………….….4  Dental procedure too invasive………….….5  Dental procedure will make me gag………….….6  Reminds me of the experience of assault……….….7  No reason………….….8  No response………….….99 |  |
| --- | --- | --- | --- |
| Q602 | Do you prefer a male or female dentists? | Male ………….….1  Female ………….….2  Either ………….….3  No response………….….99 |  |
| Q603 | How would you rate your current oral health? | Very good………….….1  Good………….….2  Fair………….….3  Poor………….….4  Very poor………….….5  Do not know………….….6  No response………….….99 |  |
| Q604 | Do you have any of the following oral health problems? | Hole in tooth ………….….1  Sensitive tooth ………….….2  Bleeding gums ………….….3  Swollen gums ………….….4  Bad breath ………….….5  Fractured tooth ………….….6  Discoloured tooth ………….….7  Painful tooth ………….….8  Oral ulcers ………….….9  Missing tooth ………….….10  No tooth problem ………….….11  No response………….….99 |  |
| Q605 | Do you have any of the following challenges with your mouth? | Eating ………….….1  Speaking ………….….2  Cleaning your mouth ………….….3  Relaxing ………….….4  Smiling ………….….5  School work ………….….6  Contact with people ………….….7  Emotional state ………….….8  No response………….….99 |  |
| Q606 | Has your experience of sexual assault impacted on your willingness to visit a dental clinic? | Yes ………..1  No ………..2  No response ………..99 |  |
| Q607 | Have you experienced any negative reactions during dental treatment? | Yes ………..1  No ………..2  No response ………..99 |  |
| Q608 | Have you ever told your dentists about your sexual assault experience? | Yes ………..1  No ………..2  No response ………..99 |  |
| Q609 | Have you had a dentists ask you any sexual assault experience? | Yes ………..1  No ………..2  No response ………..99 |  |
| Q610 | Would you be willing to share with your dentists information about your sexual assault experience? | Yes ………..1  No ………..2  No response ………..99 |  |
| Q611 | Do you have any other health problems? | Yes ………..1  No ………..2  No response ………..99 |  |

**Section 7: Corah’s Dental Anxiety Scale**

**Mark your answer with x**

**701. If your child had to go to the dentist tomorrow, how would (s)he feel about it. Mark the alternative that best describes the feelings**

………. (S)He will look forward to it as a reasonably enjoyable experience

………. (S)He wouldn’t care anyway or the other

………. (S)He would be a little uneasy about it

………. (S)He would be afraid that it would be unpleasant and painful

………. (S)He would be very frightened of what the dentist might do

**702. When your child is in the waiting room in the dentist’s office for his/her turn in the chair, how would (s)he feel?**

………. Relax

………. A little uneasy

………. Tense

………. Anxious

………. So anxious (s)he may sometimes break out in sweat or almost feel physically sick

**703. When your child is in the dentist’s chair waiting while he gets his drill ready to begin working on his/her teeth, how does (s)he feel?**

………. Relax

………. A little uneasy

………. Tense

………. Anxious

………. So anxious (s)he may sometimes break out in sweat or almost feel physically sick

**704. Your child is in the dentist’s chair to have his/her teeth cleaned. While (s)he is waiting and the dentists is getting out the instruments which will be used to scrape his/her teeth around the gun, how does (s)he feel?**

………. Relax

………. A little uneasy

………. Tense

………. Anxious

………. So anxious (s)he may sometimes break out in sweat or almost feel physically sick

**Section 8: General Health Questionnaire**

| Name | | | | Date | |
| --- | --- | --- | --- | --- | --- |
| Please consider the last four weeks and answer the following questions by selecting and circling one of the four answer options. | | | | | |
| Question | 1 | 2 | 3 | | 4 |
| 801. Been able to concentrate on what you’re doing | Better than usual | Same as usual | Less than usual | | Much less than usual |
| 802. Lost much sleep over worry | Not at all | No more than usual | Rather more than usual | | Much more than usual |
| 803. Felt you were playing a useful part in things | More so than usual | Same as usual | Less useful than usual | | Much less useful |
| 804. Felt capable of making decisions about things | More so than usual | Same as usual | Less useful than usual | | Much less useful |
| 805. Felt constantly under strain | Not at all | No more than usual | Rather more than usual | | Much more than usual |
| 806. Felt you couldn’t overcome your difficulties | Not at all | No more than usual | Rather more than usual | | Much more than usual |
| 807. Been able to enjoy your normal day-to-day activities | More so than usual | Same as usual | Less useful than usual | | Much less useful |
| 808. Been able to face up to your problems | More so than usual | Same as usual | Less useful than usual | | Much less useful |
| 809. Been feeling unhappy and depressed | Not at all | No more than usual | Rather more than usual | | Much more than usual |
| 810. Been losing confidence in yourself | Not at all | No more than usual | Rather more than usual | | Much more than usual |
| 811. Been thinking of yourself as a worthless person. | Not at all | No more than usual | Rather more than usual | | Much more than usual |
| 812. Been feeling reasonably happy, all things considered | More so than usual | About the same as usual | Less so than usual | | Much less than usual |

**Section 9: Patient Health Questionnaire (PHQ-9)**

| No. | **Questions and filters** | Coding categories | Skip to |
| --- | --- | --- | --- |
| Over the last 2 weeks, how often have you been bothered about any of the following problems | | | |

| Q901 | Little interest or pleasure in doing things | Not at all………….….0  Several days………….….1  More than half the days………….….2  Nearly every day………….….3  No response………….….99 |  |
| --- | --- | --- | --- |
| Q902 | Feeling down, depressed or hopeless | Not at all………….….0  Several days………….….1  More than half the days………….….2  Nearly every day………….….3  No response………….….99 |  |
| Q903 | Trouble falling or staying asleep, or sleeping too much | Not at all………….….0  Several days………….….1  More than half the days………….….2  Nearly every day………….….3  No response………….….99 |  |
| Q904 | Feeling tired or having little energy | Not at all………….….0  Several days………….….1  More than half the days………….….2  Nearly every day………….….3  No response………….….99 |  |
| Q905 | Poor apetite or overeating | Not at all………….….0  Several days………….….1  More than half the days………….….2  Nearly every day………….….3  No response………….….99 |  |
| Q906 | Feeling bad about yourself – or that you are a failure or have let your family down | Not at all………….….0  Several days………….….1  More than half the days………….….2  Nearly every day………….….3  No response………….….99 |  |
| Q907 | Trouble concentrating on things such as reading or watching the television | Not at all………….….0  Several days………….….1  More than half the days………….….2  Nearly every day………….….3  No response………….….99 |  |
| Q908 | Moving or speaking so slowly that other people could have noticed. Or the opposite – being so figety or restless that you have been moving around a lot more than usual. | Not at all………….….0  Several days………….….1  More than half the days………….….2  Nearly every day………….….3  No response………….….99 |  |
| Q909 | Thoughts that you would be better off dead, or off hurting yourself | Not at all………….….0  Several days………….….1  More than half the days………….….2  Nearly every day………….….3  No response………….….99 |  |
| Q910 | If you checked off any problems, how difficult has these problems made it for you to do your work, take care of things at home, or get along with other people? | Not difficult at all………….….  Somewhat difficult………….….  Very difficult ………….….  Extremely difficult………….…. |  |

**Section 10: Use of psychoactive substances**

| **No.** | | **Questions and Filters** | | **Coding categories** | | | | | **Skip to** | |
| --- | --- | --- | --- | --- | --- | --- | --- | --- | --- | --- |
| PLEASE TELL THE RESPONDENT]  I am going to ask you some sensitive and personal questions. Your answers are completely confidential andwill not be divulged to anyone | | | | | | | | | | |
| Q1001 | | | Some people take alcohol, others don’t. how frequently do you take drinks containing alcohol?  [SINGLE CODE ONLY] | | Every day…………………. 1  At least once a week……….. 2  Less than once a week…… 3  Never…………………….. 4  Not Sure………………….. 8  No response………………. 99 | | | | |  |
| Q1002 | | Some people have tried a range of different types of Psychoactive drugs **(Drugs that make a person feel high**). Which of the following, if any, have you tried?    **[READ OUT; PROBE FOR OTHERS; MULTIPLE CODES POSSIBLE]** | |  | Yes | No | No response | |  |  |
|  |  |  |  | Marijuana | 1 | 2 | 9 | |  |  |
|  |  |  |  | Glue (Solution) | 1 | 2 | 9 | |  |  |
|  |  |  |  | Cocaine | 1 | 2 | 9 | |  |  |
|  |  |  |  | Heroin | 1 | 2 | 9 | |  |  |
|  |  |  |  | Others specify ( ] ….1 | | | | |  |  |
| Q1003 | | Some people have tried injecting cocaine or heroin using a syringe and needle. Have you done this in the last 12 months?  **[Drugs injected for medical purposes or treatment of an illness do not count]** | | Yes ….. …………………..1  No. ….. .……………2  No response ……………..9 | | | | |  |  |
| Q1004 | | In your last school examination, how will you rate your academic performance? Were you amongst the ……… | | Top 10% in your set…………….1  Top 20% in your set……………….2  Top 50% in your set……………….3  Top 75% in your set………………..4  Last 25% in your set…………………5  Cannot rate myself……………….6  No response…………………99 | | | | |  |  |

**Section 11: Suicide Behaviour Questionnaire – Revised (SBQ-R)**

| No. | Questions and Filters | Coding categories | Skip to |
| --- | --- | --- | --- |
| Q1101 | Have you ever thought about killing yourself? | Never ………….….1  It was just a brief passing thought………….….2  I have had a plan once to kill myself but did not try to do it .3  I had a plan at least once to kill myself and really wanted to die .4  No response………….….99 | If yes go to 1102 |
| Q1102 | Have you ever attempted to kill yourself? | I have attempted to kill myself, but did not want to die ………..1  I have attempted to kill myself, and really hoped to die ………..2  No response………….….99 |  |
| Q1103 | How often have you thought about killing yourself in the past year? | Never ………….….1  Rarely (1 time)………….….2  Sometimes (2 times) ………. .3  Often (3-4 times)…………… .4  Very Often (5 or more times )…………… .5  No response………….….99 |  |
| Q1104 | Have you ever told anyone that you were going to commit suicide, or that you might do it? | No ………….….1  Yes at one time but did not really want to die………….….2  Yes at one time but really want to die ………. .3  Yes more than once but did not really want to die…………… .4  Yes more than once but really want to die …………… .5  No response………….….99 |  |
| Q1105 | How likely is it that you will attempt suicide one day | Never ………….….1  No chance at all………….….2  Rather unlikely ………. .3  Unlikely…………… .4  Likely…………… .5  Rather likely ………….….6  Very likely ………….….6  No response………….….99 |  |

**Section 12: Mental Health Issues**

| No. | Questions and Filters | Coding categories | Skip to |
| --- | --- | --- | --- |
| Q1201 | Diagnosis as in the case note | schizophrenia………….….1  depression………….….2  bipolar disorder………….….3  epilepsy………….….4  Drug misuse………….….5  anxiety………….….6  others………….….99 |  |
| Q1202 | Duration of current episode | Less than 6 months ………..1  More than 6 months ………..2  No response ………..99 |  |
| Q1203 | Number of medications | one………..1  two ………..2  more than two………..3  No response ………..99 |  |
| Q1204 | Have you experienced side effects of these medications? | Yes ………..1  No ………..2  No response ………..99 |  |
| Q1205 | Number of episodes | one………..1  two ………..2  more than two………..3  No response ………..99 |  |
| Q1206 | Have you ever thought about killing yourself? | Yes ………..1  No ………..2  No response ………..99 |  |
| Q1207 | Have you ever attempted to kill yourself? | Yes ………..1  No ………..2  No response ………..99 |  |

**THANK YOU VERY MUCH FOR YOUR TIME.**

**[NOTE: INTERVIEWERS EXPLAIN THAT SOMEONE WILL BE VISITING SUBSEQUENTLY AND PLEASE REMEMBER TO TURN TO THE NEXT PAGE.]**

**INFORMATION SHEET FOR STUDY PARTICIPANTS**

**Sexual, reproductive, mental and oral health needs of adolescents in Southern Nigeria: a case for integrated approach to adolescent health care management**

If you would like more information, have personal concerns, call  07062920394 send a mail to toyinukpong@yahoo.co.uk. The study is being undertaken by the Adolescent Health Working Group, Nigeria

**Introduction:** This project is being implemented by a team of researchers from the Obafemi Awolowo University, University of Ibadan, University of Port-harcourt, Babcock University, University of Benin and University of Nigeria, Enugu. The study is being led by Dr MO Ukpong. We would like to ask you and your child some questions to find out about certain oral health practices that children engage in and the reasons why. We would also be able to learn about his/her mental health status and sexual and reproductive health.

**Purpose of this study:** This study will try to find out if there is any relationship between oral habits, mental health and sexual and reproductive health. We would like to learn if dentists can also be actively involved in working with adolescents on mental health and sexual and reproductive health issues.

**Procedures:** Persons working on this project will ask you some questions and fill the questionnaire. They will ask you questions about your child’s age, work, feeding and how he/she takes care of his/her mouth. Other questions we will ask include things that you think may make the child fearful, anxious or depressed, and questions about the awareness and use of sexual and reproductive health services. We would also look at the mouth and hand to look for signs of holes in the teeth and gum problems. All examination in the mouth will be done with gloves and sterile mouth mirrors.

**Benefits:** You will not experience any direct benefit from participation; you may however benefit in the future from information learned from this study.

**Risks**: There are no risks involved in participating in this study. The only discomfort you may have with this study will be the time spent in filling the questionnaire and slight discomfort during intra-oral examination by investigators.

**Compensation:** You will not be paid any compensation for participating in this study.

**Confidentiality:** All study procedures will be conducted in private, and every effort will be made to protect your privacy and confidentiality. Your name will not be written on your questionnaire, and will never be used in connection with any of the information given. Serial numbers will be used. All information we get from you will be stored securely and will only be released to investigators

**Respondents’ Rights:** Your child will only take part in this study if both you and your child agree to participate and sign the consent form. You are also free not to continue with the study if you wish by telling any of the investigators of your decision. There will be no punishment involved if you wish to withdraw from the study.

**Conflict of Interest**: We declare that there are no conflicts of interest.

**CONSENT FORM**

**Sexual, reproductive, mental and oral health needs of adolescents in Southern Nigeria: a case for integrated approach to adolescent health care management**

***Subject’s Agreement/Consent Form:***

I have read the information provided above, or it has been read to me.

I have had the opportunity to ask questions about it and any questions I have asked have been answered to my satisfaction. I consent voluntarily to participate in this study and understand that I have the right to withdraw from the study at any time.

**Yes No**

**----------------------------------------------------------------------------------------------------**

Signature/Thumb print of Research Respondent. Date:

Printed Name of Research Subject’s Legal Guardian

Signature/thumb print of Person Obtaining Consent. Date:

Printed Name of Person Obtaining Consent.

**ASSENT FORM**

**Sexual, reproductive, mental and oral health needs of adolescents in Southern Nigeria: a case for integrated approach to adolescent health care management**

***Subject’s Agreement/Assent Form:***

I have read the information provided above, or it has been read to me.

I have had the opportunity to ask questions about it and any questions I have asked have been answered to my satisfaction. I consent voluntarily to permit my Child/Ward to participate in this study and understand that I have the right to withdraw my Child/Ward from the study at any time.

**Yes No**

**----------------------------------------------------------------------------------------------------**

Signature/Thumb print of Research Respondent. Date:

Printed Name of Research Subject’s Legal Guardian

Signature/thumb print of Person Obtaining Consent Date:

Printed Name of Person Obtaining Consent.

**DATA COLLECTION SHEET**

**Anthropometric measurements**

1. Weight: ………………. kg

2. Height: ……………….. m

**OHI-S (Simplified) - (Greene and Vermillion, 1964)**

**Criteria for calculating Plaque score**

The presence of plaque is verified on the buccal surface of 6 index teeth.

6 1 6

6 1 6

0 = no plaque present

1 = plaque covering no more than 1/3 of the surface in question.

2 = plaque covering more than 1/3, but no more than 2/3 of the surface.

3 = plaque covering more than 2/3 of the surface.

**Only fully erupted teeth are scored. There is no substitution for excluded teeth.**

**Criteria for calculating Calculus score**

The presence of calculus is verified on the buccal surface of 6 index teeth.

6 1 6

6 1 6

0 = No calculus present

1 = Supragingival calculus extending only slightly below the free gingival margin (not more than 1 mm).

2 = Supragingival calculus covering more than one third but not more than two thirds of the exposed tooth surface OR the presence of individual flecks of subgingival calculus around the cervical portion of the tooth or both.

3 = Supragingival calculus covering more than two thirds of the exposed tooth surface Or a continuous heavy band of subgingival calculus around the cervical portion of the tooth or both.

**Oral hygiene score for individual** = Debris+ calculus scores

Number of examined surfaces

1. – 1.2 = Good, 1.3 - 3.0 = Fair, > 3.1 = Poor

**PLAQUE INDEX (PI) (Silness and Loe, 1964)**

The six index teeth are

6 2 4

4 2 6

Scoring Criteria

0 = No plaque

1 = A film of plaque adhering to the free gingival margin and adjacent area of the tooth, which cannot be seen with the naked eye. But only by using disclosing solution or by using probe.

2 = Moderate accumulations of deposits within the gingival pocket, on the gingival margin and/ or adjacent tooth surface, which can be seen with the naked eye.

3 = Abundance of soft matter within the gingival pocket and/or on the tooth and gingival margin.

**PI SCORE FOR INDIVIDUAL**:

**GINGIVAL INDEX (GI) (Loe and Silness, 1963)**

The six index teeth are

6 2 4

4 2 6

The examination is done by a blunt probe. Partially erupted teeth, retained roots, teeth with periapical lesion and third molars should be excluded and there is no substitution.

**Scoring Criteria**

0 = No inflammation.

1 = Mild inflammation. Slight change in color, slight edema, no bleeding on probing.

2 = Moderate inflammation. Moderate glazing, redness, bleeding on probing.

3 = Severe inflammation. Marked redness and hypertrophy, ulceration, tendency to spontaneous bleeding.

**Gingiva Index score for Individual** = Total scores

Number of surfaces examined

0.1 -- 1 mild gingivitis; 1.1 -- 2   moderate gingivitis; 2.1 – 3 severe gingivitis

1. **Teeth present**

2. **Caries status using dmft/DMFT**:

| 55 | 54 | 53 | 52 | 51 | 61 | 62 | 63 | 64 | 65 |
| --- | --- | --- | --- | --- | --- | --- | --- | --- | --- |
|  |  |  |  |  |  |  |  |  |  |

| 18 | 17 | 16 | 15 | 14 | 13 | 12 | 11 | 21 | 22 | 23 | 24 | 25 | 26 | 27 | 28 |
| --- | --- | --- | --- | --- | --- | --- | --- | --- | --- | --- | --- | --- | --- | --- | --- |
|  |  |  |  |  |  |  |  |  |  |  |  |  |  |  |  |

|  |  |  |  |  |  |  |  |  |  |  |  |  |  |  |  |
| --- | --- | --- | --- | --- | --- | --- | --- | --- | --- | --- | --- | --- | --- | --- | --- |
| 48 | 47 | 46 | 45 | 44 | 43 | 42 | 41 | 31 | 32 | 33 | 34 | 35 | 36 | 37 | 38 |

|  |  |  |  |  |  |  |  |  |  |
| --- | --- | --- | --- | --- | --- | --- | --- | --- | --- |
| 85 | 84 | 83 | 82 | 81 | 71 | 72 | 73 | 74 | 75 |

3. **Caries status using pufa/PUFA**:

| 55 | 54 | 53 | 52 | 51 | 61 | 62 | 63 | 64 | 65 |
| --- | --- | --- | --- | --- | --- | --- | --- | --- | --- |
|  |  |  |  |  |  |  |  |  |  |

| 18 | 17 | 16 | 15 | 14 | 13 | 12 | 11 | 21 | 22 | 23 | 24 | 25 | 26 | 27 | 28 |
| --- | --- | --- | --- | --- | --- | --- | --- | --- | --- | --- | --- | --- | --- | --- | --- |
|  |  |  |  |  |  |  |  |  |  |  |  |  |  |  |  |

|  |  |  |  |  |  |  |  |  |  |  |  |  |  |  |  |
| --- | --- | --- | --- | --- | --- | --- | --- | --- | --- | --- | --- | --- | --- | --- | --- |
| 48 | 47 | 46 | 45 | 44 | 43 | 42 | 41 | 31 | 32 | 33 | 34 | 35 | 36 | 37 | 38 |

|  |  |  |  |  |  |  |  |  |  |
| --- | --- | --- | --- | --- | --- | --- | --- | --- | --- |
| 85 | 84 | 83 | 82 | 81 | 71 | 72 | 73 | 74 | 75 |

**O’sullivan index for measurement of dental erosion:**

The central incisors, lateral incisors, and first molars in the upper and lower jaws will be examined. The index of O’Sullivan (O’Sullivan *et al* 1998) will be adopted to record the distribution, severity, and amount of affected teeth.

6 2 1 1 2 6

6 2 1 1 2 6

**Site on erosion on each tooth**

Code A: Labial or buccal only

Code B: Lingual or palatal only

Code C: Occlusal or incisal only

Code D: Labial and incisal/occlusal

Code E: Lingual and incisal/occlusal

Code F: Multi-surface

**Grade of severity (worst score for an individual tooth recorded)**

Code 0: Normal enamel

Code 1: Matt appearance of the enamel surface with no loss of contour

Code 2: Loss of enamel only (loss of surface contour)

Code 3: Loss of enamel with exposure of dentine (enamel-dentin junction visible)

Code 4: Loss of enamel and dentine beyond enamel dentine junction

Code 5: Loss of enamel and dentine with exposure of the pulp

Code 9: Unable to assess (e.g. tooth crowned or large restoration)

**Area of surface affected by erosion**

Code -: Less than half of surface affected

Code +: More than half of surface affected

**ORTHODONTIC EXAMINATION**

| Teeth present |  |  |  |  |
| --- | --- | --- | --- | --- |
| 1. Facial profile |  | Straight | Convex | Concave |
| 2. Lips |  | Competent | Potentially competent | Incompetent |
| 3. Jackson lip classification |  |  |  |  |
| 4. Skeletal relationship |  | Class 1 | Class 2 | Class 3 |
| 5. Overjet | ___ (mm) | Normal | Increased | Increased |
| 6. Overbite | ____ (mm) | Normal | Deep bite | Incomplete bite |
| 7. Anterior open bite | ___ (mm) | Unilateral | Bilateral |  |
| 8. Posterior open bite | ___ (mm) | Unilateral | Bilateral |  |
| 9. Canine relationship |  | Class I | Class I1 | Class I11 |
| 10. Primary second molar relationship |  | Flush | Mesial | Distal |
| 11. First Molar relationship |  | Class 1 | Class II | Class III |
| 12. Anterior cross bite | ____ (mm) | Absent | Unilateral | Bilateral |
| 13. Buccal cross bite |  | Absent | Unilateral | Bilateral |
| 14. Lingual cross bite |  | Absent | Unilateral | Bilateral |
| 15. Upper anterior inclination |  | Normal | Proclined | Retroclined |
| 16. Lower anterior inclination |  | Normal | Proclined | Retroclined |
| 17. Upper anterior crowding | --- (mm) | Mild | Moderate | Severe |
| 18. Upper anterior spacing | ------ (mm) | Absent | Present |  |
| 19. lower anterior spacing | ------ (mm) | Absent | Present |  |
